# Jintiange Capsule May Have a Positive Effect on Pain Relief and Functional Activity in Patients with Knee Osteoarthritis: A Meta-Analysis of Randomized Trials

**DOI:** 10.1155/2021/7908429

**Published:** 2021-11-02

**Authors:** Zijian Yan, Jiahao Li, Xiaoming He, Zifeng Wang, Yinuo Fan, Jiacong Xiao, Liang Mo, Sheng Wang

**Affiliations:** ^1^The First Clinical Medical College, Guangzhou University of Chinese Medicine, Number 12, Jichang Road, Baiyun District, Guangzhou 510405, Guangdong Province, China; ^2^Guangdong Research Institute for Orthopedics & Traumatology of Chinese Medicine, No. 261, Longxi Road, Liwan District, Guangzhou 510378, China; ^3^Wuxi People's Hospital Affiliated to Nanjing Medical University, Number 299, Qingyang Road, Liangxi District, Wuxi 214000, Jiangsu Province, China; ^4^Department of Orthopedics and Traumatology, Nantong TCM Hospital Affiliated to Nanjing University of Chinese Medicine, Number 41, Jianshe Road, Nantong 226001, Jiangsu Province, China

## Abstract

**Background:**

Knee osteoarthritis (KOA) occurs frequently in the elderly and causes pain, especially when they walk. Traditional Chinese medicine treatment is effective in releasing knee osteoarthritis. Jintiange (JTG) capsule is widely used in treating knee osteoarthritis, but its clinical effects such as pain relief are still unclear. This meta-analysis aims to evaluate the clinical results systematically and negative effects of JTG capsule in patients with knee osteoarthritis.

**Methods:**

A meta-analysis of clinical randomized controlled trials (RCTs) on JTG capsule treatment was carried out in KOA patients. The search time was from the establishment of the database to May 2021. The database included PubMed, Cochrane Library, EMBASE, Web of Science database, Chinese Biomedical database (CBM), Chinese VIP information, China National Knowledge Infrastructure (CNKI), and WanFang database. The outcome indicators were extracted from the included literature and analyzed, and the risk of bias was assessed through Cochrane Handbook 5.0.1.

**Results:**

Twenty-two articles analyzed in this study involved 1887 patients. JTG capsule used alone or used with other interventions affects total effective rate significantly (RR: 1.19; 95% Cl: 1.11, 1.29; *P*=0.045), VAS score (SMD: −0.74; 95% Cl: −0.90, −0.59; *P* ≤ 0.001), WOMAC score (SMD: −0.77; 95% Cl: −0.96, −0.59; *P* ≤ 0.001), and Lequesne score (SMD: −0.82; 95% Cl: −1.02, −0.61; *P*=0.010).

**Conclusion:**

Our current evidence indicated that JTG capsule may release the pain of KOA patients and improve their functional activity. However, considering the unsatisfactory quality of the included trials, more high-quality trials are needed to prove this issue.

## 1. Introduction

Osteoarthritis (OA) is a worldwide inflammatory joint disease, being one of the main causes of joint disability [1], and its incidence increases with age [2]. Knee osteoarthritis (KOA) accounts for 83% of OA. The prevalence rate of knee osteoarthritis is estimated to be 42.8% for women and 21.5% for men in China [3]. In the past, KOA was considered a cartilage degenerative disease, but now, the concept has changed to a complex condition that affects the entire joints. Cartilage, subchondral bone, synovium, and systemic inflammation are all involved in the onset of the disease [4]. Therefore, the treatment of KOA is quite complicated. Physical therapy and exercise cannot alleviate the process of KOA. In addition, the pharmacological treatment of KOA relies on analgesics such as acetaminophen, nonsteroidal anti-inflammatory drugs (NSAIDs), and intra-articular injections. However, gastrointestinal discomfort and dose dependence are common problems with these drugs [5]. Patients with severe symptoms need to undergo knee arthroplasty, posing a significant economic burden to the patient and the family [6]. Therefore, to slow down the development process of KOA, reduce the side effects of treatment, and reduce the economic burden, as an inexpensive clinical method, Chinese herbal medicine is expected to become an alternative therapy for the prevention and treatment of KOA.

Chinese herbal medicine has been used in the healthcare system to prevent and treat various diseases in China's history. Jintiange (JTG) capsule, as a kind of herbal medicine, is composed of artificial tiger bone powder, which has anti-inflammatory, bone-formation, and antiosteoporosis effects. JTG capsule has been proven to contain high calcium levels, and the ratio of calcium to phosphorus in the capsule suits for human absorption [7]. Studies showed that tiger bones could increase an individual's pain threshold and relieve joint pain [8]. Despite its wide application, at present, the efficacy and potential adverse effects of JTG capsule on KOA are still controversial.

Therefore, here, we aim to conduct a meta-analysis of the effects of JTG capsule on KOA, focusing on clinical effectiveness and drug safety.

## 2. Materials and Methods

This work was conducted as claimed by the recommendations of the Cochrane and follows the Preferred Reporting Items for Systematic Reviews and Meta-Analyses (PRISMA) guidelines [9, 10].

### 2.1. Selection of Studies

All randomized controlled trials (RCTs) investigating JTG capsule combined with other drugs or therapies in the treatment of osteoarthritis were not limited by language or publication status. However, the nonrandomized controlled trials or animal trials were excluded.

### 2.2. Selection of Participants

Patients were diagnosed with KOA through validation criteria, such as the American Rheumatism Association (ARA), American College of Rheumatology (ACR), the Kellgren Lawrence classification (KL), and radio-graphic evidence [11]. However, secondary KOA caused by rheumatoid, bone tuberculosis, trauma, endocrine diseases, and the other reasons that affected bone metabolism were not analyzed by this research. Patients with previous knee joint infection, knee deformity before adulthood, unequal length of lower limbs, history of knee joint trauma surgery, and history of knee joint tumors were also not involved.

### 2.3. Types of Interventions

The experimental group was treated with JTG capsule alone or combined with conventional medication for intervention. The treatment dose was three times a day and three capsules each time, and the treatment duration was four to twelve weeks. The control group was treated with conventional Western medicine alone. Besides, the treatment of the two groups was carried out at the same time.

### 2.4. Types of Outcome Measures

According to the author's definition, the main result is the total effective rate. Effective: joint swelling and pain are significantly alleviated, joint activity improves, the patient feels better, and related inspection indicators are basically restored; invalid: joint swelling and pain, joint activity, patient self-feeling, and related inspection indicators are basically not significantly improved, or even worse. The second result includes VAS score, WOMAC score, Lequesne score, and the incidence of adverse events during treatment.

### 2.5. Search Strategy

Two researchers systematically conducted electronic searches in the following databases: PubMed, Cochrane Library, EMBASE, Web of Science database, Chinese Biomedical Database (CBM), Chinese VIP Information, China National Knowledge Infrastructure (CNKI), and WanFang, while the searches were accomplished from the inception of each database to 1 May 2021. During the process, if the two researchers disagree, the third researcher would make the decision. The search strategy of PubMed was as follows, and we adjusted it when searching other Chinese or English databases: (jintiange capsule OR jin tian ge capsule OR jintiange jiaonang OR jin tian ge jiaonang) AND (osteoarthritis, knee OR gonarthrosis OR osteoarthrosis OR osteoarthrit^*∗*^ OR osteoarthropathy OR arthralgia).

The two researchers also manually searched the reference lists of all identified articles for possible related studies to supplement the relevant literature. Integration and deletion of duplicate trials were performed on the EndNote software.

### 2.6. Data Extraction and Quality Assessment

The two researchers extracted relevant data and characteristics from the study, including the researcher, year of publication, sample size, male to female ratio, mean age of patients, intervention, control, duration of treatment, and outcome measures, and then investigated into it. The third author was responsible for resolving conflicts in the process. The quality of the study was independently evaluated by two researchers regarding the Cochrane Handbook for Systematic Reviews of Interventions [12]. The evaluation criteria were as follows: random sequence generation, allocation concealment, blinding of participants and personnel, blinding of outcome assessments, incomplete outcome data, selective reporting, and other bias. Meanwhile, the two reviewers routinely classify each study as low risk, high risk, or unclear. If there are disagreements, the result would refer to the third researcher's point of view.

### 2.7. Statistical Analysis

This meta-analysis was conducted by using Review Manager (RevMan) and Stata SE-64 (computer program). Regarding the research results, the relative risk with a 95% confidence interval was used for binary variables, and the weighted average difference and 95% confidence interval were utilized for continuous variables. I^2^ were employed to test the heterogeneity of the study. Due to clinical and methodological factors, there was likely to be a high degree of heterogeneity. Thus, even if I^2^ was small, this meta-analysis would use a random-effect model. The funnel chart and Begg's test were employed to test for potential publication bias. In addition, a sensitivity analysis was performed through sequential deletion tests to check the stability of the main results.

## 3. Result

### 3.1. Search Results

**Figure 1:**
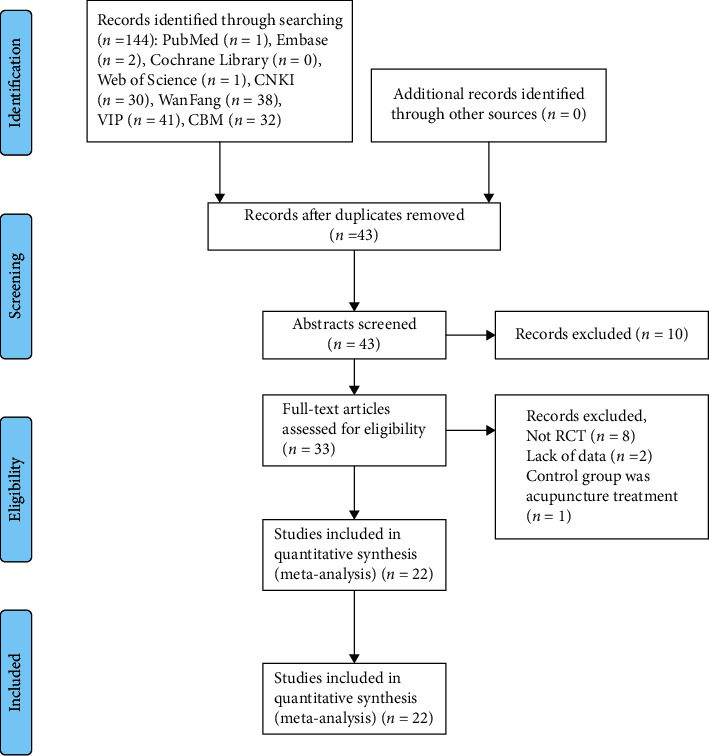
The inclusion process of the literature.

Acting by the search strategy, 144 references were identified. After excluding duplicate studies, 43 studies were scanned based on their abstracts and titles. Then, 33 articles were evaluated by full text. After the full manuscript was assessed, ten records were excluded with the following reasons: not RCT (*n* = 8), lack of outcomes (*n* = 2), and the control group was treated with acupuncture (*n* = 1). Eventually, 22 studies were included in this meta-analysis (Table 1). The PRISMA statement flow chart shows this process (Figure 1).

A total of 1887 participants were randomized into experimental groups (*n* = 943) and control groups (*n* = 944). The sample size ranged from 56 to 130. The ethnicity of all participants was Chinese. Moreover, all the studies enrolled KOA patients.

### 3.2. Risk of Bias Assessment

**Figure 2:**
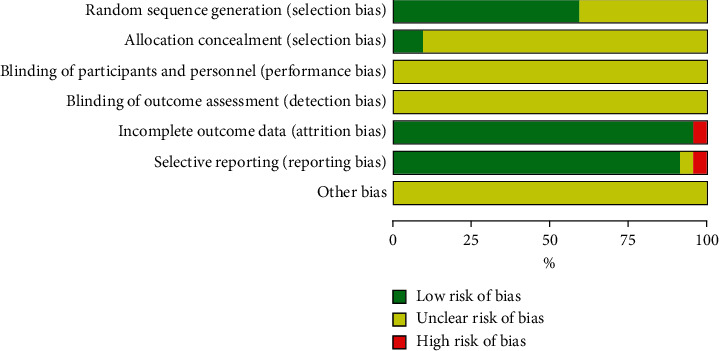
Risk of bias assessment in studies.

**Figure 3:**
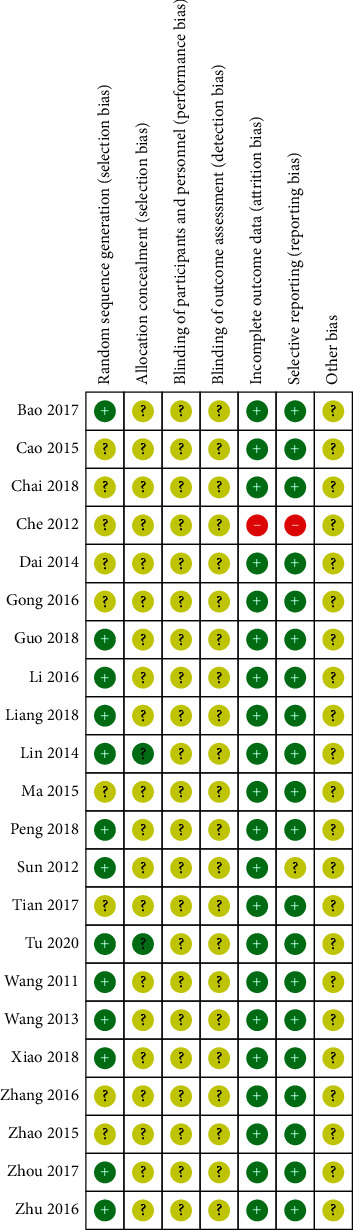
Risk of bias assessment for each included study in the review.

In general, the methodological quality of the included trials may not be high enough (Figures 2 and 3). All of the 22 included studies involved two-arm designs and were declared as random controlled trials, and 13 trials reported proper generation methods (random number table or coin toss) with a low risk of bias [13, 15, 16, 19–21, 23, 28, 30]. Nine trials did not describe the randomization procedure clearly [14, 17, 18, 22, 29, 31–34]. Only two trials reported the concealed allocation method of patients and investigators [13, 25]. In the incomplete outcome and the selective outcome reporting, a test was judged as high risk because the observation indicators in the test were not shown in the results [33]. None of the trials reported any blinding of patients and investigators.

### 3.3. Primary Outcomes

#### 3.3.1. Total Effective Rate

**Figure 4:**
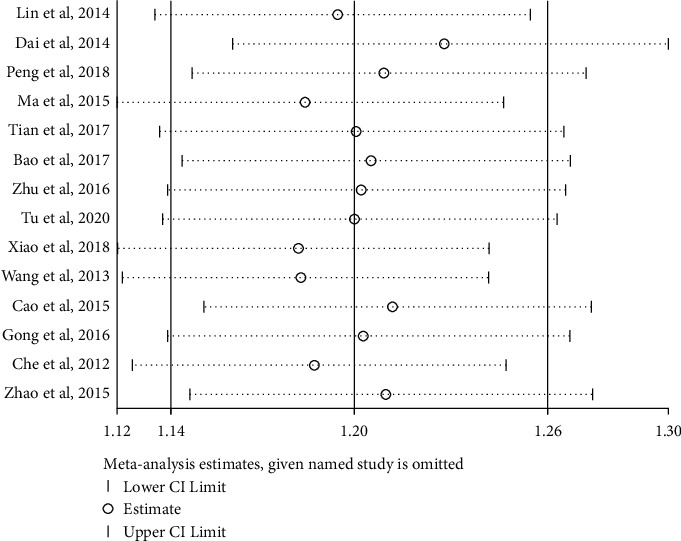
Sensitivity analysis of the total effective rate for each included study in the review.

**Figure 5:**
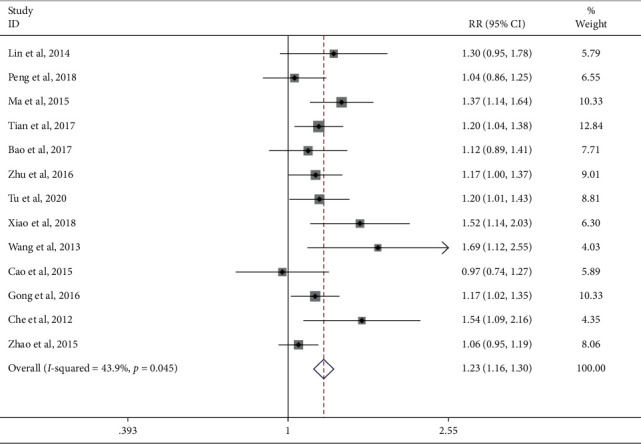
Forest plot of JTG capsule vs. conventional therapies on the total effective rate.

Fourteen studies reported the total effective rate of the JTG capsule group and the Western medicine group. Meta-analysis showed that the total effective rate of the JTG capsule group was significantly higher (RR: 1.19; 95% Cl: 1.07, 1.33; *P* ≤ 0.001, *I*^2^ = 83.4%) than that of the Western medicine group. The results of all these trials showed high heterogeneity, and thus, a sensitivity analysis was conducted (Figure 4), which showed that the included trail [14] had a more significant impact on the results. A careful review of the included article found that this article only included women with postmenopausal knee osteoarthritis, which may cause higher heterogeneity. The remaining 13 articles were used to analyze the total effective rate and get a new result (RR: 1.19; 95% Cl: 1.11, 1.29; *P*=0.045, *I*^2^ = 43.9%, Figure 5).

#### 3.3.2. VAS Score

**Figure 6:**
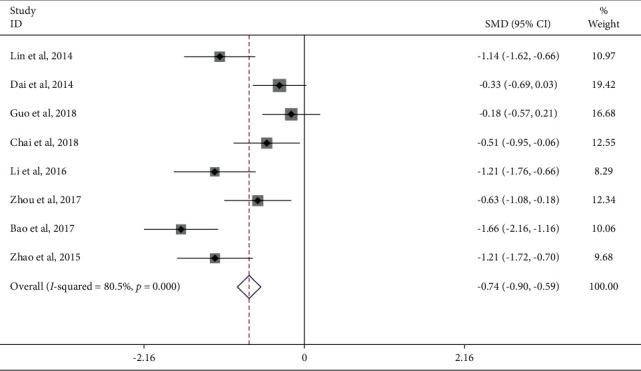
Forest plot of JTG capsule vs. conventional therapies on VAS score.

Results on the VAS score were presented in eight trials involving 671 KOA patients. Meta-analysis showed that the VAS score of the JTG capsule group was significantly lower (SMD: −0.74; 95% Cl: −0.90, −0.59; *P* ≤ 0.001, *I*^2^ = 80.5%, Figure 6) than that of the Western medicine group. Subgroup analysis (Supplementary Figure 1) was performed for the mean baseline of sample size ≥80 and <80. In 3 studies [13, 19, 34], sample size baseline levels were, respectively, 78, 60, and 70, which in the remaining 5 studies were all higher than 80. The heterogeneity analysis suggested that there was lower heterogeneity after subgroup analysis. The results suggested that the sample size may be a source of heterogeneity.

#### 3.3.3. WOMAC Score

**Figure 7:**
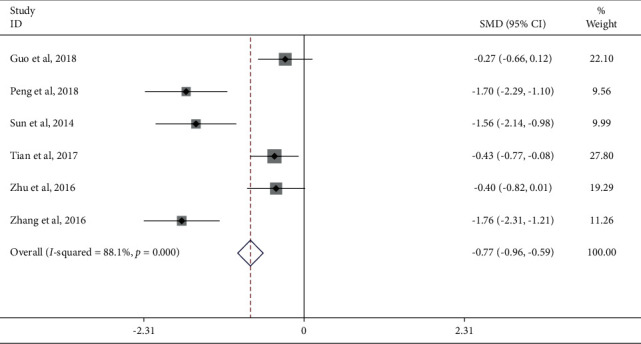
Forest plot of JTG capsule vs. conventional therapies on WOMAC score.

Compared with the Western medicine group, six studies reported the WOMAC score. Meta-analysis showed that the WOMAC score of the JTG capsule group was significantly lower (SMD: −0.77; 95% Cl: −0.96, −0.59; *P* ≤ 0.001, *I*^2^ = 88.1%, Figure 7) than that of the Western medicine group. Subgroup analysis (Supplementary Figure 2) was performed for the mean baseline of age ≥60 and <60. In 2 studies [15, 24], age baseline levels were, respectively, 66.58 and 68.63, which in the remaining 4 studies were all lower than 60. The heterogeneity analysis suggested that there was lower heterogeneity after subgroup analysis. The results suggested that the age may be a source of heterogeneity.

#### 3.3.4. Lequesne Score

**Figure 8:**
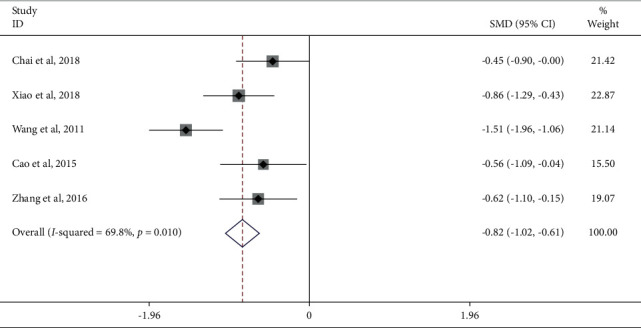
Forest plot of JTG capsule vs. conventional therapies on the Lequesne score.

Five studies reported the Lequesne score of the JTG capsule group and the Western medicine group. Meta-analysis showed that the Lequesne score of the JTG capsule group was significantly lower (SMD: −0.82; 95% Cl: −1.02, −0.61; *P*=0.010, *I*^2^ = 69.8%, Figure 8) than that of the Western medicine group. The large heterogeneity may be due to the small number of trials reporting this indicator, which suggested that the results of the Lequesne score were unstable and need to be interpreted with caution.

#### 3.3.5. Adverse Effect

Of the 24 trials, only three trials involved adverse events related to the treatment of KOA with JTG capsules. Few patients experienced some mild stomach discomfort, such as nausea and bloating.

#### 3.3.6. Publication Bias

**Figure 9:**
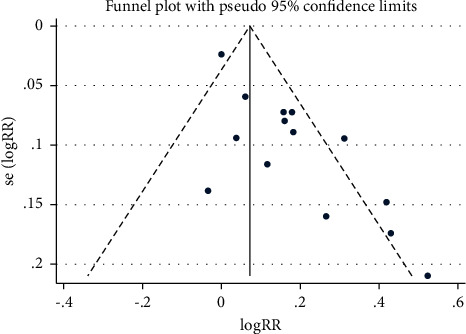
Funnel plot of the total effective rate.

Although the funnel plot (Figure 9) of the total effective rate was asymmetrically distributed, Begg's test showed no potential publish bias (*P*=0.125).

## 4. Discussion

Traditional herbal medicine has been used as a complementary and alternative treatment option for patients with osteoarthritis for a long time. JTG capsule may improve the function of the knee joint to a certain extent and has a particular analgesic effect [35]. However, its efficacy and side effects in treating knee osteoarthritis are uncertain. To our knowledge, this is the first meta-analysis of the efficacy and side effects of JTG capsule on KOA.

Compared with the Western medicine group, the overall estimate showed that the symptoms of KOA were significantly relieved in 14 randomized controlled trials after four to twelve months. In terms of the VAS score, eight studies showed that the pain level of KOA patients was significantly reduced after four weeks of treatment. Similarly, in terms of the WOMAC score, six studies showed that, after four weeks of treatment, the WOMAC score of the JTG capsule group was lower than that of the Western medicine group, which indicated that it effectively reduced the patient's pain, stiffness, and the difficulty in activities in daily life. In terms of the Lequesne score, five studies showed that the pain level of KOA patients was significantly reduced, and the function of walk of patients was improved after treatment. However, the heterogeneity of some outcome indicators is high. The main reason may be that the number of trails investigated is limited. Therefore, more large-scale clinical trials are needed to prove our results better in the future. The side effect of JTG capsule may be mild stomach upset. In general, our research results showed that JTG capsule could reduce the pain of KOA patients, thereby improving the knee joint function of the patients, and there was no apparent liver and kidney damage except for mild gastrointestinal reactions. Part of the included trials also performed serological tests on patients, and the results showed that IL-1 and IL-6 in the JTG capsule group were lower than those in the Western medicine group. Therefore, it was supposed that the Jintiange capsule had a specific therapeutic effect on the inflammatory response of knee osteoarthritis. Because of the insufficient number of trials, we did not conduct a statistical analysis of inflammation-related indicators.

Clinically, JTG capsule is usually used to treat many common orthopaedic diseases such as osteoporosis, fractures, and rheumatoid arthritis. In 2017, it was listed as an effective treatment method in the treatment guidelines for osteoporotic fractures in China [36]. However, there is no large randomized controlled trial to prove that JTG capsule has an apparent effect on knee osteoarthritis. Ping [37] conducted acetic acid writhing and electric shock on mice tails to test the analgesic effect of tiger bone, one of the main ingredients of JTG capsule, and the results showed that it could effectively relieve pain. Se et al. [38] also demonstrated that tiger bone could enhance the pain threshold and prolong the latent period of pain response by conducting the hot plate test and the acetic acid writhing test on mice. JTG capsule is an oral medication that has the advantage of good compliance and safety, with no apparent effect on hepatic and renal function [39]. Pharmacological studies have shown that JTG capsule can regulate the expression of osteopontin and matrix metalloproteinase 3, thereby affecting the metabolism of articular cartilage and subchondral bone, and can effectively improve the symptoms of postmenopausal osteoarthritis [40]. Modern pharmacological studies have shown that artificial tiger bone is rich in calcium, which can improve bone toughness and increase bone density. At the same time, the artificial tiger bone is rich in many factors required for bone growth, which can provide adequate nutrition for chondrocytes, improve cartilage cell metabolism, and inhibit degenerative diseases of the human body. The pharmacology of the active ingredient of JTG capsule, artificial tiger bone, and natural tiger bone is basically the same, and the safety is higher. Therefore, the adverse reaction rate of the observation group is low [7].

To sum up, our research showed that the JTG capsule could reduce the pain of KOA patients, which is expected to become an optional treatment for KOA, and may be related to the inhibition of inflammatory factors IL-1 and IL-6. However, in order to verify this mechanism, more experiments are expected.

## 5. Limitations

Unavoidably, the comprehensive analysis of all studies in this research conducted had some limitations, which should be recognized. First, due to unclear allocation concealment, blinding of participants and personnel, and blinding of outcome assessments, some of the included studies may be of average quality. Second, although this research aimed to conduct an unbiased literature search without language restriction, all the experiments in this review were held in China and published in Chinese. There were no relevant foreign experiments, which may lead to potential prejudice, thus limiting the representativeness of this research. Third, few studies mentioned adverse reactions during or after treatment. Besides, no trials reported long-term follow-up, so the long-term safety of the intervention is still unknown.

## 6. Conclusions

This meta-analysis showed that the JTG capsule may have effects on KOA in the following aspects: relief of pain and improvement of functional activity. However, no conclusions about other indicators or safety issues could be drawn from the available evidence. Higher-quality and more rigorous research on larger samples are expected to confirm current results.

## Figures and Tables

**Table 1:** The basic characteristics of the included studies.

Trail	Sample size (T/C)	Man/woman	Age (y), mean ± SD or median (range)	T	C	Duration (weeks)	Main outcomes
Jian and Sheng [13]	78 (39/39)	30/48	55.39 ± 8.38	JTG capsules	Alendronate	12	①②
Yi and Huan [14]	120 (60/60)	0/120	56 (48–60)	JTG capsules	Glucosamine hydrochloride	6	①②
Jianli [15]	102 (51/51)	NR	66.58 ± 6.23	JTG capsules	Glucosamine hydrochloride	6	②③
Jiewei et al. [16]	60 (30/30)	19/41	53.46 ± 5.74	JTG capsules	Etocoxib	12	①③
Fang and Qing [17]	80 (40/40)	NR	66.31 ± 5.67	JTG capsules	Glucosamine sulfate	12	②④
Cunzhu and Xiaodong [18]	120 (60/60)	61/59	61 (45–70)	JTG capsules + C	Sodium hyaluronate	5	①
Yuhong et al. [19]	60 (30/30)	27/33	54.96 ± 10.55	JTG capsules + C	Sodium hyaluronate	24	②
Yiebi et al. [20]	80 (40/40)	NR	52.0 ± 6.4	JTG capsules	Loxoprofen sodium	12	②
Zhilin [21]	120 (60/60)	NR	72.6 ± 10.2	JTG capsules	Voltaren	4	⑤
Fang et al. [22]	130 (65/65)	47/83	55.52 ± 5.43	JTG capsules	Diclofenac sodium	8	①③
Yun et al. [23]	83 (41/42)	31/52	44.9 ± 6.3	JTG capsules + C	Meloxicam	12	①②
Junlian and Pengcheng [24]	90 (45/45)	25/65	68.63 (50–75)	JTG capsules	Aceclofenac	12	①③
Dahua et al. [25]	90 (45/45)	42/48	59.8 ± 1.4	JTG capsules	Glucosamine sulfate	4	①
Dongwei [26]	90 (45/45)	41/49	60.8 ± 7.1	JTG capsules	Piroxicam	12	①④
Jinping at al. [27]	98 (49/49)	48/50	56.9 ± 13.0	JTG capsules + C	Naproxen	14	④
Guoyu [28]	72 (36/36)	38/34	66 (60–86)	JTG capsules	Ibuprofen	12	①
Jiangang et al. [29]	60 (30/30)	14/46	61.57 ± 6.68	JTG capsules	Aceclofenac	4	①④
Jian et al. [30]	60 (30/30)	16/44	56.85	JTG capsules	Voltaren	4	③
Yunzhao and Lin [31]	100 (50/50)	43/57	62.2 (40–80)	JTG capsules	Glucosamine hydrochloride	12	①
Dongliang et al. [32]	72 (36/36)	23/49	58.25 (44–75)	JTG capsules	Glucosamine hydrochloride	12	③④
Tao et al. [33]	61 (31/30)	26/35	51.3	JTG capsules	Glucosamine hydrochloride	12	①
Shuangli et al. [34]	70 (35/35)	27/43	62.12 (42–71)	JTG capsules	Glucosamine hydrochloride	12	①②

T: trial group; C: control group; NR: not reported; ①: the total effective rate; ②: the VAS score; ③: the WOMAC score; ④: the Lequesne score; ⑤: adverse events. Mean ± SD is mean, and median (range) is median.

## Data Availability

The datasets supporting the conclusions of this study are included within the article.

## Supplementary Materials

Supplementary Materials Subgroup analysis of VAS score was performed for the mean baseline of sample size ≥80 and <80 (Supplemental Figure 1). Subgroup analysis of WOMAC score was performed for the mean baseline of age ≥60 and <60 (Supplemental Figure 2).
