# Supplementary figures and images for: Jintiange Capsule May Have a Positive Effect on Pain Relief and Functional Activity in Patients with Knee Osteoarthritis: A Meta-Analysis of Randomized Trials
Source: Evid Based Complement Alternat Med. 2021 Nov 2;2021:7908429. doi: 10.1155/2021/7908429 (PMC8577893; doi:10.1155/2021/7908429)

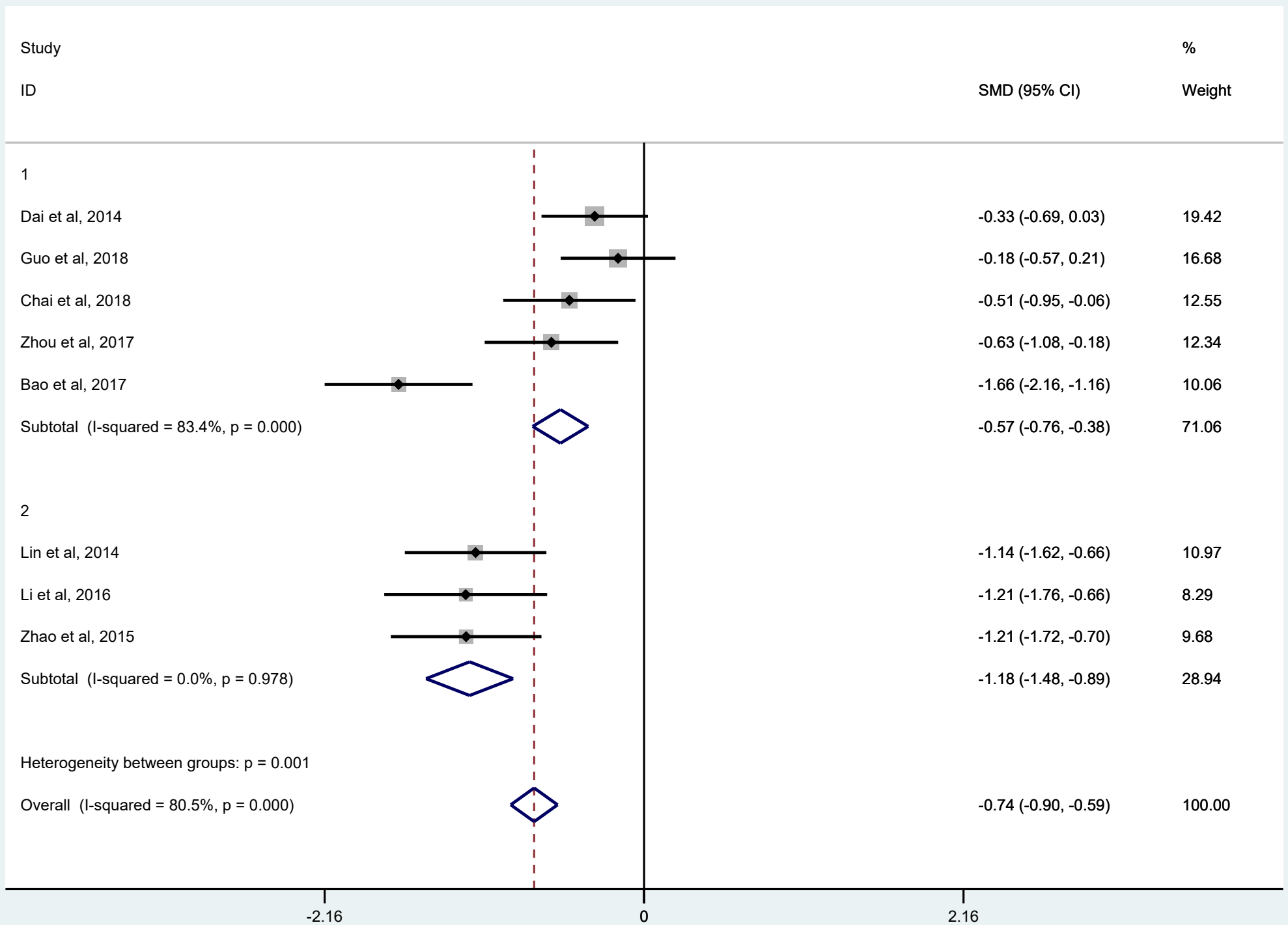

Supplement: Supplementary Materials — Subgroup analysis of VAS score was performed for the mean baseline of sample size ≥80 and <80 (Supplemental Figure 1). Subgroup analysis of WOMAC score was performed for the mean baseline of age ≥60 and <60 (Supplemental Figure 2). [file 7908429.f1.zip › 7908429.f1/Supplemental Figure 1 (1).pdf]

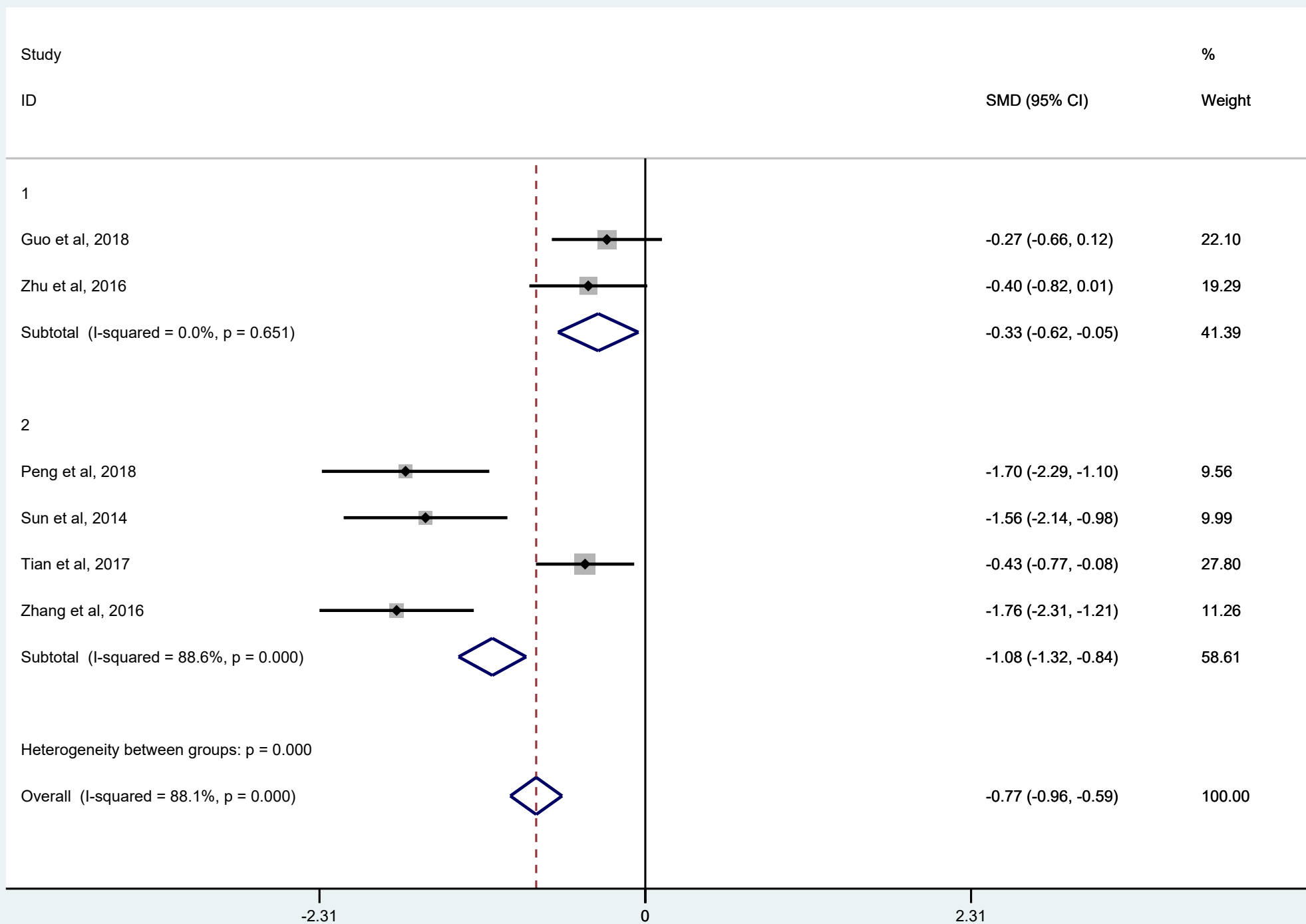

Supplement: Supplementary Materials — Subgroup analysis of VAS score was performed for the mean baseline of sample size ≥80 and <80 (Supplemental Figure 1). Subgroup analysis of WOMAC score was performed for the mean baseline of age ≥60 and <60 (Supplemental Figure 2). [file 7908429.f1.zip › 7908429.f1/Supplemental Figure 2 (1).pdf]
